# Do ethnic inequalities in multimorbidity reflect ethnic differences in socioeconomic status? The HELIUS study

**DOI:** 10.1093/eurpub/ckz012

**Published:** 2019-02-14

**Authors:** Wim J G M Verest, Henrike Galenkamp, Bea Spek, Marieke B Snijder, Karien Stronks, Irene G M van Valkengoed

**Affiliations:** 1 Department of Public Health, Amsterdam UMC, Amsterdam Public Health (APH) Research Institute, University of Amsterdam, Amsterdam, The Netherlands; 2 Department of Clinical Epidemiology, Biostatistics and Bioinformatics, Amsterdam UMC, University of Amsterdam, Amsterdam, The Netherlands

## Abstract

**Background:**

The burden of multimorbidity is likely higher in ethnic minority populations, as most individual diseases are more prevalent in minority groups. However, information is scarce. We examined ethnic inequalities in multimorbidity, and investigated to what extent they reflect differences in socioeconomic status (SES).

**Methods:**

We included Healthy Life in an Urban Setting study participants of Dutch (*N* = 4582), South-Asian Surinamese (*N* = 3258), African Surinamese (*N* = 4267), Ghanaian (*N* = 2282), Turkish (*N* = 3879) and Moroccan (*N* = 4094) origin (aged 18–70 years). Educational level, employment status, income situation and multimorbidity were defined based on questionnaires. We described the prevalence and examined age-adjusted ethnic inequalities in multimorbidity with logistic regression analyses. To assess the contribution of SES, we added SES indicators to the age-adjusted model.

**Results:**

The prevalence of multimorbidity ranged from 27.1 to 53.4% in men and from 38.5 to 69.6% in women. The prevalence of multimorbidity in most ethnic minority groups was comparable to the prevalence among Dutch participants who were 1–3 decades older. After adjustment for SES, the odds of multimorbidity remained significantly higher in ethnic minority groups. For instance, age-adjusted OR for multimorbidity for the Turkish compared to the Dutch changed from 4.43 (3.84–5.13) to 2.34 (1.99–2.75) in men and from 5.35 (4.69–6.10) to 2.94 (2.54–3.41) in women after simultaneous adjustment for all SES indicators.

**Conclusions:**

We found a significantly higher prevalence of multimorbidity in ethnic minority men and women compared to Dutch, and results pointed to an earlier onset of multimorbidity in ethnic minority groups. These inequalities in multimorbidity were not fully accounted for by differences in SES.

## Introduction

European populations are ageing and the prevalence of long-term disorders and the number of patients facing multiple chronic diseases are increasing.^1^^–^^3^ Multimorbidity, often defined as having two or more chronic diseases,^3^^–^^5^ is associated with a decreased quality of life, higher psychological distress and a higher mortality.^3^ In addition, it leads to a rise in the utilization of specialist physician services, hospital admissions, post-operative complications and a higher cost of care.^3^^,^^6^ The expected rise in prevalence of multimorbidity in the forthcoming years is increasingly being recognized as a major challenge for public health and health care, for instance because interventions directed towards patients with a single disease may not suit those with multimorbidity.^2^^,^^3^ To enable policy and practice to adequately target populations at high risk of multimorbidity, understanding the variation in the multimorbidity burden across subgroups in the population is of the utmost relevance.

Evidence shows that the burden of multimorbidity may differ across subgroups within the population. Inequalities in the occurrence of multimorbidity by age and sex have been described.^7^^–^^9^ In addition, studies in the USA have shown inequalities between ethnic groups, mostly to the disadvantage of African American and Hispanic vs. white Americans.^7^^,^^10^ While populations in Europe are increasingly becoming ethnically diverse, studies examining the inequalities in multimorbidity in ethnic minority vs. majority European populations are scarce. The prevalence of multimorbidity is likely to be higher in ethnic minority populations as compared to the majority population. Indeed, one study^11^ has described ethnic inequalities in multimorbidity in a selected UK population. Moreover, multiple studies in other contexts that focus on the prevalence of single chronic diseases, such as diabetes and stroke, indicate that ethnic minority groups have a higher prevalence of disease than the majority population and often already at a younger age.^12^ In addition, in coming years a larger increase in the burden of disease may be expected for ethnic minority populations in comparison with the majority population, as has been shown for type 2 diabetes in Amsterdam.^13^ To be able to adequately target populations with complex health problems, it seems crucial to fill the gap in the knowledge on ethnic inequalities in multimorbidity.

In general, ethnic minority groups have a lower socioeconomic status (SES) than the majority population.^14^ Minorities may experience linguistic and cultural barriers or discrimination that reduce the chance of getting a job, education or housing.^15^ Such differences in SES may contribute to ethnic inequalities in chronic diseases^8^ and—thus—in multimorbidity.^16^ Previous studies have found a significant negative association between SES and the prevalence of chronic diseases and multimorbidity.^2^^,^^9^^,^^17^ In addition, higher rates of multimorbidity were found to be particularly common in deprived socioeconomic areas.^18^^,^^19^ However, in line with the scarcity of data on European ethnic minority populations,^20^ the extent to which ethnic inequalities in multimorbidity reflect differences in SES has not been investigated.

We used data from the multi-ethnic Healthy Life in an Urban Setting (HELIUS) study^21^ to examine the prevalence of multimorbidity among 18–70-year old men and women of Dutch, South-Asian Surinamese, African Surinamese, Ghanaian, Turkish and Moroccan origin living in the same city in the Netherlands. In addition, we analyzed the extent to which ethnic inequalities in multimorbidity reflect differences in SES, defined by educational level, employment status and income situation.

## Methods

### Study design and study population

This study is a cross-sectional analysis of the baseline data from the HELIUS study.^16^ Baseline data collection took place in 2011–15 and included individuals aged 18–70 years from different origins residing in Amsterdam: Dutch, Surinamese, Ghanaian, Moroccan and Turkish origin. Subjects were randomly sampled, stratified by ethnic origin, from the municipality register of Amsterdam.^16^^,^^21^

Ethnicity was defined before data collection according to the country of birth of the participant as well as that of their parents, as registered in the municipality register. After data collection, Surinamese were further classified according to self-reported ethnic origin, into ‘African Surinamese’, ‘South-Asian Surinamese’, ‘Javanese Surinamese’ and ‘other/unknown Surinamese’ (see Supplementary appendix S1 for further information).

HELIUS was able to contact 55% of those invited, and 50% of those contacted agreed to participate (60% among Dutch, 51% among Surinamese, 61% among Ghanaians, 41% among Turkish and 43% among Moroccan origin). Non-response analyses showed only small differences in SES between participants and non-participants.^21^

The data were collected by means of a questionnaire, a physical examination, and by obtaining biological samples. Participants could choose to fill in the questionnaire on paper or online. Participants unable to fill in a questionnaire were offered assistance from a trained (ethnically matched) interviewer. The HELIUS study was conducted in accordance with the Declaration of Helsinki and has been approved by the AMC Ethical Review Board. All participants provided written informed consent.

We included the 23 942 participants with completed questionnaires. Of these, Javanese Surinamese (*n* = 250), other/unknown Surinamese (*n* = 286) and those with another/unknown ethnic origin (*n* = 50) were excluded (Supplementary appendix S1). Subsequently, we also excluded 259 participants in whom the presence or absence of multimorbidity could not be determined. After successive exclusion based on missing information on educational level (*N* = 190), income situation (*N* = 364) and employment status (*N* = 181), 22 362 participants remained of Dutch (*N* = 4582), South-Asian Surinamese (*N* = 3258), African Surinamese (*N* = 4267), Ghanaian (*N* = 2282), Turkish (*N* = 3879) and Moroccan (*N* = 4094) origin.

### Measures

Multimorbidity was defined as having two or more chronic diseases.^4^^,^^5^ The presence of these chronic diseases was determined from a list of 20 predefined diseases that a participant reported to have had in the 12 months prior to the baseline measurement, either diagnosed by a doctor or not (Supplementary appendix S2). In addition to these, we considered the presence of ‘depressed mood’ based on the score of the PHQ-9 (sum score ≥10) as an additional chronic disorder.^22^ If the presence of multimorbidity could not be determined because of missing values on one or more diseases (i.e. those with only one reported disease and at least one missing item, and those with zero reported diseases and at least two missing items), multimorbidity was coded as missing.

For descriptive purpose, age was classified into five groups (18–30; 31–40; 41–50; 51–60 and 61–70 years). SES was defined by educational level, employment status and perceived income situation. Educational level was based on the highest qualification attained, either in the Netherlands or in the country of origin, and was categorized into (i) *higher education* (higher vocational schooling or university), (ii) *medium education* (intermediate vocational schooling or intermediate/secondary schooling), (iii) *lower education* (lower vocational schooling or lower secondary schooling) and (iv) *no or elementary education.* Employment status was categorized in (i) *having a paid job*, (ii) *not in the work force* (retired/studying/homemaking), *unemployed* (unemployed and looking for work/social benefit recipient) and (iii) *incapacitated (*unable to work). Perceived income situation was measured as whether participants experienced problems managing their household income: (i) *no problems* (no problems at all), (ii) *no real problems* (no problems, but I have to watch what I spend), (iii) *some problems* and (iv) *lots of problems.*

### Statistical analysis

The association between ethnicity and chronic diseases may differ for men and women.^24^^,^^25^ As this seemed to be the case in our study (data not shown), we performed all further analyses in men and women separately.

First, chi-square tests were used to assess univariate differences between ethnic groups in the prevalence of multimorbidity. We estimated associations of SES parameters with multimorbidity in each ethnic group with age-adjusted logistic regression analyses. Because differences in the association of SES with single chronic diseases have been reported between ethnic groups,^2^^,^^17^ we also formally tested the consistency of the association across ethnic groups with a multiplicative interaction term. Second, age-adjusted ethnic differences in multimorbidity were examined using logistic regression analyses. Third, to examine the extent to which ethnic inequalities in multimorbidity reflect differences in SES, we examined the change in the estimates of the ethnic differences after adding each SES indicator separately, and all SES indicators simultaneously, to the age-adjusted logistic regression model. Finally, we verified in an additional analysis if consideration of interaction between SES indicators and ethnicity changed interpretation regarding ethnic differences in multimorbidity.

All analyses were performed using SPSS version 24 (IBM Corp. Released 2016. IBM SPSS Statistics for Windows, Version 24.0. Armonk, NY: IBM Corp.). A level of *P* < 0.05 was considered statistically significant.

## Results

### Characteristics

The mean age was around 45 years in Dutch, South-Asian Surinamese, African Surinamese and Ghanaian men and women, while Turkish and Moroccan participants were ∼5 years younger (table 1). The Dutch were most often highly educated, while the Ghanaian group had the highest proportion of participants with low or no education. Most men had paid work, while this was lower in women. Income problems were least observed in the Dutch, and most often in Turkish participants. The median number of chronic diseases reported ranged from 1 to 2 in men and from 1 to 3 in women.

**Table 1:** Characteristics of the population, by sex and ethnic origin

Men (*n* = 12 812)	Dutch (*n* = 2473)	South-Asian Surinamese (*n* = 1745)	African Surinamese (*n* = 2540)	Ghanaian (*n* = 1392)	Turkish (*n* = 2127)	Moroccan (*n* = 2535)
Age, years						
Mean (sd)	46.9 (13.8)	44.5 (13.7)	47.5 (13.3)	46.3 (12.0)	40.2 (12.4)	41.7 (12.9)
Age groups (%)						
18–30	16.1	21.5	15.4	13.9	26.7	23.4
31–40	19.9	15.2	13.4	11.9	21.5	23.8
41–50	19.3	26.1	21.0	30.6	28.5	25.8
51–60	24.0	24.2	34.4	36.5	19.1	18.7
61–70	20.6	13.0	15.8	7.1	4.3	8.3
^a^Educational level (%)						
High	59.5	23.3	17.8	9.3	15.5	18.6
Medium	23.5	31.5	34.8	30.4	29.7	33.5
Low	13.7	32.8	41.1	45.2	30.9	22.4
No or very low	3.4	12.3	6.4	15.1	23.9	25.5
^b^Employment status (%)						
Paid work	75.4	64.7	62.4	70.4	68.0	64.5
Not in working force	16.0	11.7	11.2	7.2	7.2	7.7
Unemployed	5.9	15.1	18.7	17.0	15.9	17.3
Incapacitated	2.8	8.5	7.6	5.4	8.9	10.5
^c^Income situation (%)						
No problems	48.4	36.5	26.1	33.7	20.4	23.3
No real problems	35.1	31.6	33.6	28.7	25.4	31.7
Some problems	12.8	22.2	26.1	24.2	28.8	26.7
Lots of problems	3.7	9.7	14.1	13.5	25.5	18.2
Number of diseases						
Median [IQR]	1 [0–2]	2 [0–4]	1 [0–3]	1 [0–2]	2 [0–4]	1 [0–3]

Women (*N* = 9550)	Dutch (*n* = 2109)	South-Asian Surinamese (*n* = 1513)	African Surinamese (*n* = 1727)	Ghanaian (*n* = 890)	Turkish (*n* = 1752)	Moroccan (*n* = 1559)
Age, years						
Mean (sd)	45.3 (14.2)	45.6 (13.3)	47.5 (12.3)	43.1 (10.9)	39.7 (12.4)	38.8 (13.0)
Age groups (%)						
18–30	21.4	17.6	12.4	13.9	26.9	32.6
31–40	16.2	15.1	14.1	21.6	23.2	23.3
41–50	21.4	26.2	26.7	36.6	29.1	22.0
51–60	23.6	28.2	33.3	25.6	16.3	17.0
61–70	17.5	13.0	13.6	2.4	4.6	5.1
^a^Educational level (%)						
High	61.4	22.0	26.0	4.7	13.9	16.6
Medium	20.8	28.9	37.1	22.0	28.9	34.6
Low	14.6	33.8	32.2	36.5	20.2	15.2
No or very low	3.2	15.3	4.7	36.9	37.0	33.6
^b^Employment status (%)						
Paid work	72.4	57.8	62.4	52.6	39.9	38.4
Not in working force	18.6	16.7	12.6	7.7	36.9	39.7
Unemployed	5.3	15.5	14.8	28.4	14.0	15.1
Incapacitated	3.4	10.0	10.2	11.3	9.2	6.8
^c^Income situation (%)						
No problems	40.8	26.7	21.8	24.6	17.6	21.2
No real problems	38.9	32.2	33.9	36.3	26.8	37.4
Some problems	15.3	24.8	27.4	24.7	31.5	26.1
Lots of problems	5.0	16.3	16.9	14.4	24.2	15.3
Number of diseases						
Median [IQR]	1 [0–2]	2 [1–5]	2 [1–4]	1 [0–3]	3 [1–6]	2 [1–4]

^a^Educational level: High = higher vocational schooling or university; Medium = intermediate vocational schooling or intermediate/secondary schooling; Low = lower vocational schooling or lower secondary schooling; No or very low = no or elementary schooling only.

^b^Employment status: Paid work; Not in working force = (retired/studying/homemaking); Unemployed = unemployed and seeking work/in welfare; Incapacitated = unable to work.

^c^Income situation: No problems = no, no problems at all; No real problems = no problems, but I have to watch what I spend; Some problems = yes, some problems; Lots of problems = yes, lots of problems.

### Prevalence of multimorbidity

**Figure 1:**
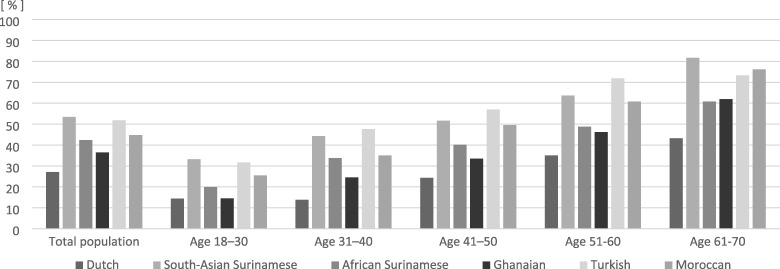
Prevalence of multimorbidity in women, by age and ethnicity

In all ethnic groups, women and higher age groups had a higher prevalence of multimorbidity than men and younger age groups (Supplementary appendix S3; figure 1). In both men (27.1%) and women (38.5%), the Dutch had the lowest prevalence of multimorbidity. The highest prevalence was found among the South-Asian Surinamese in men (53.4%) and among the Turkish in women (69.6%). In all age groups, the prevalence of multimorbidity was higher among most ethnic minority groups than among the Dutch. Figure 1 illustrates for women, that the prevalence of multimorbidity in the ethnic minority groups was often comparable with the prevalence among Dutch participants who were about 1–3 decades older. This pattern was comparable to that in men (Supplementary appendix S4). For example, the rate of multimorbidity in 61–70-year-old Dutch women (57.4%) was comparable to the prevalence among Turkish women (62.8%) aged 31–40 years. In 61–70-year-old South-Asian Surinamese men and Turkish women, the prevalence of multimorbidity was as high as 81.7 and 90.7%, respectively.

### Association of SES with multimorbidity across ethnic groups

We found consistent patterns of higher odds of multimorbidity in lower SES groups after adjustment for age in men and women in all ethnic groups (table 2). Statistical testing of effect modification revealed that the association of educational level (both men: *P* = 0.018 and women: *P* < 0.0001), employment status (men only: *P* = 0.006) and income situation (men only: *P* < 0.0001) with multimorbidity was somewhat stronger in the Dutch than in the other ethnic groups (further data not shown). Because of the consistent pattern of higher odds of multimorbidity in lower vs. higher SES groups, we adjusted for SES indicators without consideration of interaction in the main analyses of ethnic inequalities in multimorbidity.

**Table 2:** The age adjusted association between SES indicators and multimorbidity in men and women by ethnicity

	Dutch	South-Asian Surinamese	African Surinamese	Ghanaian	Turkish	Moroccan
	OR (95% CI)	OR (95% CI)	OR (95% CI)	OR (95% CI)	OR (95% CI)	OR (95% CI)
Men
Educational level^a^						
High	ref	ref	ref	ref	Ref	ref
Medium	1.72 (1.34–2.19)	1.63 (1.22–2.17)	1.58 (1.17–2.12)	1.83 (1.04–3.23)	1.89 (1.38–2.59)	1.39 (1.02–1.90)
Low	2.41 (1.83–3.19)	1.78 (1.34–2.38)	1.57 (1.18–2.09)	1.40 (0.81–2.42)	2.05 (1.50–2.79)	1.22 (0.87–1.71)
Very low or no	4.91 (2.93–8.22)	2.03 (1.38–2.99)	2.00 (1.26–3.17)	1.42 (0.76–2.64)	3.07 (2.19–4.30)	2.06 (1.48–2.87)
Employment status^b^						
Paid work	ref	ref	ref	ref	ref	ref
Not in working force	1.22 (0.93–1.59)	1.38 (0.97–1.96)	1.12 (0.80–1.56)	0.92 (0.49–1.74)	0.53 (0.34–0.82)	1.26 (0.83–1.92)
Unemployed	1.99 (1.35–2.94)	3.36 (2.42–4.67)	1.79 (1.38–2.31)	1.66 (1.15–2.40)	1.84 (1.39–2.43)	2.31 (1.74–3.07)
Incapacitated	8.40 (4.43–15.94)	5.51 (3.30–9.20)	3.55 (2.37–5.32)	5.91 (2.91–11.98)	4.33 (2.76–6.80)	7.59 (4.79–12.01)
Income situation^b^						
No problems	ref	ref	ref	ref	ref	ref
No real problems	1.15 (1.20–1.90)	1.24 (0.96–1.61)	1.27 (0.98–1.66)	1.71 (1.18–2.50)	0.92 (0.69–1.24)	1.01 (0.74–1.37)
Some problems	2.85 (2.12–3.83)	3.22 (2.38–4.36)	1.53 (1.16–2.03)	2.58 (1.76–3.79)	1.60 (1.20–2.13)	2.52 (1.85–3.43)
Lots of problems	4.48 (2.76–7.29)	4.81 (3.11–7.44)	2.58 (1.85–3.60)	2.79 (1.77–4.38)	3.64 (2.66–4.99)	5.05 (3.54–7.22)
Women
Educational level^a^						
High	ref	ref	ref	ref	ref	ref
Medium	1.77 (1.43–2.19)	1.56 (1.18–2.06)	2.23 (1.41–3.52)	1.13 (0.64–2.00)	2.20 (1.65–2.93)	1.49 (1.17–1.89)
Low	2.35 (1.84–3.01)	1.36 (1.04–1.79)	1.42 (1.15–1.77)	1.15 (0.66–1.99)	2.46 (1.80–3.36)	2.21 (1.65–2.96)
Very low or no	3.91 (2.34–6.55)	3.24 (2.18–4.83)	1.14 (0.93–1.40)	1.24 (0.72–2.15)	3.49 (1.65–2.93)	1.89 (1.47–2.45)
Employment status^a^						
Paid work	ref	ref	ref	ref	ref	ref
Not in working force	1.22 (0.98–1.53)	1.88 (1.40–2.52)	1.36 (1.05–1.78)	1.02 (0.64–1.61)	1.34 (1.08–1.65)	1.42 (1.18–1.72)
Unemployed	2.56 (1.77–3.71)	2.16 (1.59–2.93)	1.77 (1.40–2.25)	1.78 (1.38–2.29)	2.25 (1.62–3.13)	1.79 (1.38–2.31)
Incapacitated	6.36 (3.67–11.03)	5.47 (3.25–9.20)	5.89 (3.93–8.81)	3.47 (2.35–5.13)	2.99 (1.90–4.72)	5.82 (3.53–9.60)
Income situation^a^						
No problems	ref	ref	ref	ref	ref	ref
No real problems	1.68 (1.39–2.05)	1.46 (1.13–1.89)	1.53 (1.22–1.91)	0.99 (0.74–1.32)	1.15 (0.88–1.52)	1.22 (0.98–1.52)
Some problems	2.69 (2.09–3.47)	2.02 (1.52–2.69)	2.53 (2.00–3.21)	1.59 (1.16–2.17)	1.99 (1.51–2.62)	2.46 (1.92–3.16)
Lots of problems	6.82 (4.47–10.41)	3.74 (2.62–5.36)	4.70 (3.52–6.28)	2.60 (1.79–3.77)	4.54 (3.23–6.40)	3.96 (2.89–5.44)

OR = Odds Ratio, CI = Confidence Interval.

^a^Educational level: High = higher vocational schooling or university; Medium = intermediate vocational schooling or intermediate/secondary schooling; Low = lower vocational schooling or lower secondary schooling; No or very low = no or elementary schooling only.

^b^Employment status: Paid work; Not in working force = (retired/studying/homemaking); Unemployed = unemployed and seeking work/in welfare; Incapacitated = unable to work.

^c^Income situation: No problems = no, no problems at all; No real problems = no problems, but I have to watch what I spend; Some problems = yes, some problems; Lots of problems = yes, lots of problems.

### The role of SES in the association between ethnicity and multimorbidity

After adjustment for age, the OR for multimorbidity in men varied from 1.66 (1.40–1.98) in Ghanaian and 4.43 (3.84–5.13) in Turkish vs. Dutch men, and from 1.63 (1.42–1.87) in Ghanaian to 5.35 (4.69–6.10) in Turkish vs. Dutch women (table 3). The associations between ethnicity and multimorbidity persisted after additional adjustment for different SES indicators, although the strength of the associations somewhat decreased. Even adjusted for all SES indicators simultaneously, the odds of multimorbidity remained significantly higher in ethnic minority groups, except in Ghanaians. Finally, consideration of the interaction between ethnicity and SES indicators did not substantially change our results (Supplementary appendix S5). The inequalities in multimorbidity as compared to the Dutch were strengthened in the higher SES categories for some of the ethnic minority groups. In contrast to the main analyses, we also observed a difference between Dutch and Ghanaians in the highest SES groups when taking interaction into account.

**Table 3:** The contribution of SES indicators to the ethnic inequalities in multimorbidity in men and women

	Dutch	South-Asian Surinamese	African Surinamese	Ghanaian	Turkish	Moroccan
		OR (95%CI)	OR (95%CI)	OR (95%CI)	OR (95%CI)	OR (95%CI)
Men
Adjusted for age	ref	3.87 (3.34–4.49)	2.05 (1.78–2.36)	1.66 (1.40–1.98)	4.43 (3.84–5.13)	3.05(2.63–3.54)
Adjusted for age and SES-parameters separately						
Educational level	ref	3.09 (2.65–3.60)	1.63 (1.41–1.89)	1.21 (1.01–1.45)	3.20 (2.74–3.74)	2.22 (1.90–2.60)
Employment status	ref	3.37 (2.90–3.91)	1.74 (1.50–2.01)	1.47 (1.23–1.75)	3.76 (3.24–4.36)	2.46 (2.11–2.86)
Income situation	ref	3.37 (2.90–3.91)	1.60 (1.38–1.84)	1.32 (1.11–1.58)	2.92 (2.51–3.40)	2.21 (1.89–2.57)
Adjusted for age and all SES-parameters simultaneously						
	ref	2.74 (2.34–3.21)	1.31 (1.13–1.53)	1.06 (0.88–1.28)	2.34 (1.99–2.75)	1.66 (1.41–1.96)
Women						
Adjusted for age	ref	3.02 (2.64–3.45)	2.16 (1.92–2.43)	1.63 (1.42–1.87)	5.35 (4.69–6.10)	3.71 (3.28–4.20)
Adjusted for age and SES-parameters separately						
Educational level	ref	2.37 (2.06–2.72)	1.81 (1.60–2.04)	1.05 (0.90–1.22)	3.69 (3.20–4.26)	2.63 (2.29–3.00)
Employment status	ref	2.67 (2.34–3.06)	1.94 (1.72–2.19)	1.26 (1.09–1.46)	4.41 (3.85–5.05)	3.03 (2.67–3.44)
Income situation	ref	2.52 (2.20–2.89)	1.71 (1.52–1.93)	1.32 (1.14–1.52)	3.91 (3.41–4.48)	3.02 (2.66–3.42)
Adjusted for age and all SES-parameters simultaneously						
	ref	2.08 (1.81–2.40)	1.49 (1.32–1.69)	0.90 (0.76–1.05)	2.94 (2.54–3.41)	2.25 (1.96–2.58)

OR = Odds Ratio, CI = Confidence Interval.

## Discussion

### Main findings

We found large ethnic differences in the prevalence of multimorbidity (men 27.1–53.4%, women 38.5–69.6%). Compared to Dutch men and women, those of South-Asian Surinamese, African Surinamese, Ghanaian, Turkish and Moroccan origin had a higher prevalence of multimorbidity. The prevalence of multimorbidity in most ethnic minority groups was comparable with that among Dutch participants who were about 1–3 decades older. Ethnic inequalities in multimorbidity only in part reflected differences in SES indicators. After adjustment for SES indicators, the odds of multimorbidity remained significantly higher in ethnic minority men and women than in the Dutch.

### Limitations

This study has several limitations that merit discussion. First, as in most previous work on multimorbidity, we conceived multimorbidity as having two or more chronic diseases, which is relevant as a consistent predictor of morbidity and mortality.^3^ This definition does, however, not consider severity, complexity and duration of the diseases. Given the high burden of multimorbidity in ethnic minority populations, a more accurate picture may be obtained with other conceptualizations of multimorbidity, such as complex multimorbidity (three or more chronic conditions affecting at least three different body systems).^26^ Second, the age limit of 70 years in our study has resulted in large enough numbers to analyze ethnic differences in multimorbidity across age groups. However, the overall prevalence of multimorbidity might be underestimated compared to that in the general population, since the prevalence of multimorbidity increases with age.^10^ Third, we used a disease questionnaire that is used to monitor the prevalence of chronic diseases in the Netherlands, (https://www.monitorgezondheid.nl/gezondheidsmonitor-volwassenen-en-ouderen) but the use of self-reported data has several drawbacks. Disease definitions were perhaps interpreted differently across socioeconomic and ethnic groups, although the questionnaire was available in different languages. For depressed mood, however, previous research has shown that this was not the case.^27^ In addition, including all self-reports and not only those that were confirmed by a doctor has the advantage of minimizing the influence of inequalities in access to health care. However, the overall rate of multimorbidity might be overestimated as a result of this. Future studies, focussing on chronic diseases that are more objectively defined, should be performed to confirm our findings in the relevant ethnic groups in different contexts. Fourth, our measure of income situation (‘problems managing the household income’) may have reflected the perceived problems rather than the actual income situation, possibly resulting in suboptimal adjustment for actual income situation in the analyses. However, previous studies have shown that educational level is the most important marker for SES, and we used conventional ordinal measures for both educational level and employment status as well. A final limitation of our study might be that invited respondents exclude those second-generation migrants with parents from two different countries. Given the rising rate if inter-ethnic union formation, future work may strive to also include these groups.

### Discussion of the main findings

We found significant inequalities in the prevalence of multimorbidity by ethnicity; the prevalence was 1.5–5 times higher in ethnic minorities with estimates exceeding 80% in some age groups. These inequalities as compared to the Dutch are consistent with previous studies that have found a much higher prevalence of *single* chronic diseases in ethnic minority populations compared to the majority population.^28^^,^^29^ In addition, our results are in agreement with earlier studies that reported ethnic inequalities in multimorbidity.^8^^,^^11^ Of those studies, Mathur et al. was the only study that reported ethnic inequalities in the prevalence of multimorbidity in a European population. This study found, in a population with established cardiovascular morbidity, that people of South Asian and African origin had higher odds of multimorbidity than people of white European origin.^11^ Our study adds to these findings that such differences are also observed within the general population setting. In addition, those populations of Middle Eastern and North African (e.g. Turkish and Moroccan) origin also have a higher prevalence compared to a Western majority population.

Interestingly, we found that the prevalence of multimorbidity in most ethnic minority men and women was comparable to the prevalence among Dutch men and women who were 1–3 decades older. This is in line with previous reports on age differences in the occurrence of, for instance, type 2 diabetes in our population^12^ and in other studies of diabetes, cardiovascular disease and obesity.^30^^,^^31^ This implies that policy and practice should be aware that problems associated with multimorbidity, such as treatment complexity, polypharmacy and higher risk of adverse effects may already occur sooner in these populations than can be expected for the general population. Given that multimorbidity increases with age, the occurrence of these problems is likely to be even higher in older age groups than those included in our study.

Our finding of a SES gradient in multimorbidity across ethnic groups residing in the Netherlands is in accordance with previous studies that observed apparent SES gradients in the prevalence of multimorbidity in Western societies.^2^^,^^8^^,^^17^ There are likely to be several mechanisms that account for the observed relationship between SES and health.^32^ Previous research has shown that lower SES is associated with more psychosocial stress and a less healthy lifestyle,^33^ which in turn might lead to a higher risk of developing certain chronic diseases.

Despite the consistent negative association of SES with multimorbidity, controlling for SES only in part accounted for the difference in the odds for having multimorbidity in ethnic minority groups compared to the Dutch. These differences only seemed to disappear for Ghanaian men and women, although not in the highest SES groups, and remained for the other groups. The finding that the increased prevalence of multimorbidity only partially reflects the lower socio-economic status of the ethnic minority groups seems consistent with recent work by Johnson-Lawrence et al.^8^ They showed for 30–64 year old Americans that the difference in multimorbidity (two or more vs. one somatic diseases) between Non-Hispanic Blacks and Non-Hispanic whites attenuated but also persisted after controlling for SES indicators. This indicates that the inequalities in multimorbidity are likely also driven by other mechanisms. For instance, ethnic differences in lifestyle,^34^ or in psychosocial distress in relation to discrimination may affect the development of health conditions.^35^ Additionally, ethnic inequities in accessibility or quality of healthcare may play a role.^36^ This should be investigated in future studies.

## Conclusion

This study shows that the prevalence of multimorbidity is higher in men and women of Turkish, Moroccan, South-Asian Surinamese, African Surinamese and Ghanaian origin than in those of Dutch origin. While SES is associated with multimorbidity in all ethnic groups, our findings clearly indicate that the ethnic inequalities in multimorbidity persist after taking into account the lower SES of ethnic minority groups. Certain ethnic minority groups, regardless of their SES, have a high multimorbidity burden. Moreover, multimorbidity occurs 1–3 decades earlier in ethnic minority groups compared to the Dutch, implying that the adverse effects of multimorbidity in these populations may occur sooner than can be expected for those of Dutch origin. Further exploration of which diseases cluster in ethnic groups across the lifespan seems relevant to be able to target treatment guidelines and prevention efforts to specific disease patterns in the different populations at high risk.

## Supplementary Material

ckz012_Supplementary_Appendix
