# Supplementary material for: Do ethnic inequalities in multimorbidity reflect ethnic differences in socioeconomic status? The HELIUS study
Source: Eur J Public Health. 2019 Feb 14;29(4):687–93. doi: 10.1093/eurpub/ckz012 (PMC6660190; doi:10.1093/eurpub/ckz012)
Supplement: ckz012_Supplementary_Appendix [file ckz012_supplementary_appendix.docx]

**Appendix 1. Classification of ethnicity in the HELIUS study**

Ethnicity was defined according to the country of birth of the participant as well as that of their parents, as registered in the municipality register(1, 2). A participant was considered to be of non-Dutch ethnic origin if 1) he/she was born abroad and had at least one parent born abroad (first generation) or 2) if he/she was born in the Netherlands, but both parents were born abroad (second generation).

Previous research has shown a high correlation between the country of birth indicator and self-identified ethnic group indicator among Turkish, Moroccan and Surinamese people in the Netherlands(3). For the Dutch sample, people who were born in the Netherlands and whose parents were born in the Netherlands were invited.

A limitation of the country of birth indicator for ethnicity is that people who are born in the same country might have a different ethnic background, which in the Dutch context is applicable to the Surinamese population(1). Therefore, after data collection, Surinamese were further classified according to self-reported ethnic origin, into ‘African Surinamese’, ‘South-Asian Surinamese’, ‘Javanese Surinamese’ and ‘other/unknown Surinamese’.

The South-Asian Surinamese are descendants of the labourers from North India (Uttar Pradesh, Uttaranchal and West Bihar) who were indentured between 1873 and 1917. The two large migration waves of Surinamese to the Netherlands were caused mainly by the political situation in Suriname. The first wave took place at the time of the independence of Suriname in 1975; and the second wave (at the time of Desi Bouterse’s coup), in February 1980.(4)

Both South-Asian Surinamese and African Surinamese (distinction was assessed by self-report) respondents were included in this study. Together these groups form >80% of the Surinamese in the Netherlands (other groups being e.g. Chinese or Javanese). The number of participants belonging to the other subgroups were considered too small to be included in the analyses.

1. Stronks K, Snijder MB, Peters RJ, Prins M, Schene AH, Zwinderman AH. Unravelling the impact of ethnicity on health in Europe: the HELIUS study. BMC Public Health. 2013;13(1):1-10.

2. Snijder MB, Galenkamp H, Prins M, Derks EM, Peters RJG, Zwinderman AH, et al. Cohort profile: the Healthy Life in an Urban Setting (HELIUS) study in Amsterdam, The Netherlands. BMJ Open. 2017;7(12).

3. Stronks K, Kulu-Glasgow I, Agyemang C. The utility of ‘country of birth’ for the classification of ethnic groups in health research: the Dutch experience. Ethnicity & Health. 2009;14(3):255-69.

4. Choenni C, Harmsen C. Place of birth and ethnic composition of the Surinamese in the Netherlands. Bevolkingtrends. 2007;1:74-8.

**Appendix 2. List of illnesses and disorders**

“Please indicate which of the following illnesses and disorders you have now or that you have had in the past 12 months, and whether or not this was diagnosed by a doctor.

If you don’t know if you now have or if you have had a certain illness or disorder, please fill in ‘No’. Please give an answer for every illness/disorder”

1. diabetes mellitus
2. stroke, brain hemorrhage, cerebral infarction, or TIA (‘transient ischemic attack’: temporary loss of bodily function)
3. heart attack (myocardial infarction)
4. another serious heart condition (for example, heart failure or angina pectoris (severe chest pain))
5. a form of cancer (malignant disorder)
6. migraine or frequent severe headaches
7. severe or chronic fatigue
8. high blood pressure
9. narrowing of the arteries in the belly or legs (artery stenosis)
10. asthma, chronic bronchitis, lung emphysema, or CNSLD (chronic non-specific lung disease) or COPD (chronic obstructive pulmonary disease)
11. serious or persistent intestinal disorders lasting more than 3 months
12. psoriasis (a chronic skin disease)
13. eczema/chronic eczema
14. involuntary loss of urine (incontinence)
15. a serious or persistent back disorder (including slipped disc)
16. worn joints (arthrosis, osteoarthritis) in the hips and/or knees
17. chronic inflammation of the joints (inflammatory rheumatism, chronic rheumatism, rheumatoid arthritis)
18. other serious or persistent neck or shoulder disorders
19. other serious or persistent disorders of the elbow, wrist, or hand
20. chronic muscle pain or general pain throughout your whole body

**Appendix 3. Prevalence (%) of multimorbidity within ethnic groups**

|  | **Ethnic group** | | | | | |
| --- | --- | --- | --- | --- | --- | --- |
| **Men** | **Dutch** | **South-Asian Surinamese** | **African Surinamese** | **Ghanaian** | **Turkish** | **Moroccan** |
| **Total** | 27.1 | 53.4 | 42.4 | 36.4 | 51.8 | 44.8 |
| **Age, years** |  |  |  |  |  |  |
| 18–30 | 14.4 | 33.2 | 19.9 | 14.5 | 31.7 | 25.5 |
| 31–40 | 13.8 | 44.3 | 33.8 | 24.5 | 47.6 | 35,0 |
| 41–50 | 24.3 | 51.6 | 40.2 | 33.5 | 57,0 | 49.5 |
| 51-60 | 35,0 | 63.7 | 48.8 | 46.2 | 71.9 | 60.8 |
| 61-70 | 43.2 | 81.7 | 60.8 | 61.9 | 73.3 | 76.2 |
| **Educational level*** |  |  |  |  |  |  |
| High | 19.8 | 41.6 | 33.2 | 25.3 | 33.1 | 31.7 |
| Medium | 29.1 | 48.4 | 40.1 | 39.1 | 45.6 | 40,0 |
| Low | 46.5 | 61.4 | 46.7 | 36.1 | 53.2 | 41,0 |
| No or very low | 62.5 | 67.2 | 53.6 | 38.8 | 69.7 | 63.8 |
| **Employment status**** |  |  |  |  |  |  |
| Paid work | 22.2 | 44.1 | 35.3 | 32.1 | 46.8 | 34.8 |
| Not in working force | 36.8 | 56.5 | 46.4 | 25,0 | 28.6 | 43.3 |
| Unemployed | 39.5 | 72.9 | 52.3 | 46.4 | 65.6 | 57.8 |
| Incapacitated | 77.6 | 85.2 | 70.5 | 77.1 | 84,0 | 85.4 |
| **Income situation***** |  |  |  |  |  |  |
| No problems | 20.7 | 41.7 | 33.9 | 24.7 | 37.3 | 28.6 |
| No real problems | 27.7 | 46.7 | 39.4 | 34.5 | 37.5 | 31.4 |
| Some problems | 42.2 | 71.7 | 45.2 | 46.5 | 54.2 | 55.6 |
| Lots of problems | 52.6 | 77.6 | 60.2 | 51.7 | 74.9 | 72.9 |
|  |  |  |  |  |  |  |
| **Women** | **Dutch** | **South-Asian Surinamese** | **African Surinamese** | **Ghanaian** | **Turkish** | **Moroccan** |
| **Total** | 38.5 | 63.5 | 58.2 | 46.9 | 69.6 | 60.9 |
| **Age, years** |  |  |  |  |  |  |
| 18–30 | 25.5 | 46.3 | 36.8 | 17.6 | 51.9 | 43.5 |
| 31–40 | 23.7 | 51.7 | 49,0 | 31.7 | 62.8 | 58.7 |
| 41–50 | 38.4 | 61.3 | 51.9 | 51.8 | 79.8 | 70.1 |
| 51-60 | 46.5 | 73.4 | 67.1 | 65.2 | 84.7 | 77.3 |
| 61-70 | 57.4 | 83.6 | 77.7 | 84.8 | 90.7 | 88.4 |
| **Educational level*** |  |  |  |  |  |  |
| High | 30.1 | 50.3 | 52.1 | 35.4 | 45.8 | 43.4 |
| Medium | 43.3 | 60,0 | 53.5 | 42.5 | 63.4 | 53.4 |
| Low | 60,0 | 65.5 | 65.9 | 45.9 | 69.5 | 66.5 |
| No or very low | 70.9 | 84.6 | 75.6 | 52,0 | 83.5 | 74.9 |
| **Employment status**** |  |  |  |  |  |  |
| Paid work | 32.9 | 54.4 | 51.2 | 38.5 | 60.4 | 48.8 |
| Not in working force | 47.2 | 69.5 | 60.5 | 30.8 | 70.5 | 64.8 |
| Unemployed | 57.7 | 73.8 | 64.5 | 56.1 | 81.5 | 68.6 |
| Incapacitated | 80,0 | 90.2 | 88.8 | 73.9 | 87.8 | 89,0 |
| **Income situation***** |  |  |  |  |  |  |
| No problems | 29.8 | 51.7 | 44.2 | 39.8 | 53.5 | 44.8 |
| No real problems | 39.3 | 60.1 | 52.6 | 40,0 | 59.2 | 53.4 |
| Some problems | 49.3 | 69,0 | 64.4 | 52.9 | 73.4 | 72.6 |
| Lots of problems | 70.2 | 81.1 | 77.3 | 66.2 | 87.9 | 81.7 |
| Prevalence of multimorbidity are percentages  *Educational level: High= higher vocational schooling or university; Medium= intermediate vocational schooling or intermediate/secondary schooling; Low= lower vocational schooling or lower secondary schooling; No or very low= No or elementary schooling only  **Employment status: Paid work; Not in working force= (retired/studying/homemaking); Unemployed=Unemployed and seeking work/in welfare; Incapacitated=Unable to work  ***Income situation: No problems=No, no problems at all; No real problems=No problems, but I have to watch what I spend; Some problems = Yes, some problems; Lots of problems = Yes, lots of problems | | | | | | |

**Appendix 4. Prevalence of multimorbidity in men, by age and ethnicity**

**Appendix 5. Age-adjusted ethnic inequalities in multimorbidity in men and women, stratified by SES-indicators^a^**

| **MEN** | **Dutch** | **South-Asian Surinamese** | **African Surinamese** | **Ghanaian** | **Turkish** | **Moroccan** |
| --- | --- | --- | --- | --- | --- | --- |
| **Educational Level*** |  | **OR (95% CI)** | **OR (95% CI)** | **OR (95% CI)** | **OR (95% CI)** | **OR (95% CI)** |
| High | ref | 3.49 (2.68-4.53) | 1.95 (1.47-2.59) | 1.58 (0.93-2.69) | 3.06 (2.27-4.12) | 2.96 (2.21-3.97) |
| Medium | ref | 3.30 (2.50-4.36) | 1.79 (1.37-2.33) | 1.68 (1.22-2.32) | 3.36 (2.56-4.42) | 2.40(1.82-3.15) |
| Low | ref | 2.57 (1.90-3.49) | 1.27 (0.96-1.69) | 0.92 (0.67-1.26) | 2.60 (1.92-3.51) | 1.50 (1.08-2.08) |
| No or very low | ref | 1.44 (0.80-2.60) | 0.80 (0.42-1.50) | 0.46 (0.25-0.84) | 1.92 (1.12-3.29) | 1.24 (0.73-2.13) |
| **Employment status**** | | | | | | |
| Paid work | ref | 3.15 (2.63-3.76) | 1.89 (1.59-3.76) | 1.59 (1.29-1.96) | 4.16 (3.50-4.93) | 2.40 (2.00-2.87) |
| Not in working force | ref | 3.56 (2.37-5.34) | 1.74 (1.19-2.56) | 1.20 (0.62-2.32) | 1.80 (1.11-2.93) | 2.48 (1.55-3.95) |
| Unemployed | ref | 5.31 (3.30-8.54) | 1.70 (1.10-2.61) | 1.32 (0.81-2.16) | 3.83 (2.45-6.00) | 2.77 (1.78-4.34) |
| Incapacitated | ref | 2.07 (0.93-4.60) | 0.80 (0.38-1.67) | 1.12 (0.44-2.84) | 2.14 (1.00-4.60) | 2.16 (1.00-4.66) |
| **Income situation***** | | | | | | |
| No problems | ref | 3.46 (2.73-4.38) | 2.11 (1.63-2.72) | 1.37 (1.01-1.88) | 4.03 (3.06-5.31) | 2.46 (1.85-3.26) |
| No real problems | ref | 2.85 (2.21-3.67) | 1.77 (1.39-2.25) | 1.56 (1.14-2.13) | 2.46 (1.89-3.20) | 1.64 (1.27-2.13) |
| Some problems | ref | 3.92 (2.75-5.57) | 1.13 (0.83-1.55) | 1.25 (0.86-1.81) | 2.26 (1.65-3.08) | 2.17 (1.57-3.00) |
| Lots of problems | ref | 3.71 (2.02-6.83) | 1.21 (0.71-2.06) | 0.85 (0.48-1.54) | 3.27 (1.97-5.46) | 2.77 (1.62-4.72) |
| **WOMEN** | **Dutch** | **South-Asian Surinamese** | **African Surinamese** | **Ghanian** | **Turkish** | **Moroccan** |
| **Educational Level*** |  | **OR (95% CI)** | **OR (95% CI)** | **OR (95% CI)** | **OR (95% CI)** | **OR (95% CI)** |
| High | ref | 2.75 (2.17-3.48) | 2.40 (1.98-2.91) | 1.84 (1.08-3.13) | 3.01 (2.31-3.91) | 2.99 (2.37-3.76) |
| Medium | ref | 2.43 (1.87-3.15) | 1.55 (1.24-1.94) | 1.18 (0.88-1.58) | 3.74 (2.91-4.81) | 2.51 (2.00-3.17) |
| Low | ref | 1.59 (1.20-2.10) | 1.45 (1.12-1.89) | 0.90 (0.68-1.19) | 3.14 (2.31-4.27) | 2.81 (2.06-3.84) |
| No or very low | ref | 2.28 (1.24-4.17) | 1.37 (0.71-2.64) | 0.58 (0.34-0.99) | 2.68 (1.57-4.59) | 1.45 (0.85-2.45) |
| **Employment status**** | | | | | | |
| Paid work | ref | 2.50 (2.13-2.94) | 1.95 (1.69-2.25) | 1.37 (1.14-1.65) | 4.49 (3.77-5.26) | 3.02 (2.56-3.57) |
| Not in working force | ref | 3.84 (2.75-5.36) | 2.18 (1.59-2.97) | 1.14 (0.71-1.84) | 4.91 (3.79-6.35) | 3.52 (2.76-4.48) |
| Unemployed | ref | 2.11 (1.34-3.31) | 1.35 (0.89-2.05) | 0.95 (0.63-1.43) | 3.95 (2.48-6.29) | 2.11 (1.38-3.21) |
| Incapacitated | ref | 2.15 (1.03-4.51) | 1.80 (0.93-3.52) | 0.75 (0.39-1.43) | 2.11 (1.06-4.22) | 2.77 (1.34-5.72) |
| **Income situation***** | | | | | | |
| No problems | ref | 2.95 (2.33-3.73) | 1.81 (1.45-2.26) | 2.00 (1.54-2.61) | 4.91 (3.79-6.34) | 3.59 (2.85-4.53) |
| No real problems | ref | 2.55 (2.05-3.19) | 1.64 (1.35-1.99) | 1.17 (0.94-1.47) | 3.36 (2.69-4.20) | 2.60 (2.15-3.16) |
| Some problems | ref | 2.22 (1.65-2.99) | 1.70 (1.31-2.22) | 1.18 (0.87-1.60) | 3.63 (2.76-4.78) | 3.29 (2.50-4.33) |
| Lots of problems | ref | 1.62 (0.98-2.67) | 1.25 (0.79-1.98) | 0.76 (0.46-1.25) | 3.27 (2.02-5.28) | 2.09 (1.30-3.37) |

OR=Odds Ratio, CI=Confidence Interval

**^a^ Stratified results were obtained by including** interactions between ethnicity and each SES indicator in the model

P-values interaction terms SES indicators with ethnicity (men): educational level: P=0.018; employment status: P=0.006; income situation: P<0.0001
P-values interaction terms SES indicators with ethnicity (women): educational level: P<0.0001; employment status P=0.124; income situation: P=0.040

*Educational level: High= higher vocational schooling or university; Medium= intermediate vocational schooling or intermediate/secondary schooling; Low= lower vocational schooling or lower secondary schooling; No or very low= No or elementary schooling only

**Employment status: Paid work; Not in working force= (retired/studying/homemaking); Unemployed=Unemployed and seeking work/in welfare; Incapacitated=Unable to work

***Income situation: No problems=No, no problems at all; No real problems=No problems, but I have to watch what I spend; Some problems = Yes, some problems; Lots of problems = Yes, lots of problems
